# A KT intervention including the evidence alert system to improve clinician’s evidence-based practice behavior—a cluster randomized controlled trial

**DOI:** 10.1186/1748-5908-8-132

**Published:** 2013-11-13

**Authors:** Lanie Campbell, Iona Novak, Sarah McIntyre, Sarah Lord

**Affiliations:** 1 School of Medicine, University of Notre Dame Australia, corner Oxford Street and Victoria Street, Darlinghurst, NSW 2010, Australia; 2 Cerebral Palsy Alliance, PO Box 560, Darlinghurst, NSW 1300, Australia; 3 School of Medicine, University of Notre Dame Australia, corner Oxford Street and Victoria Street, Darlinghurst, NSW 2010, Australia; 4 NHMRC Clinical Trials Centre The University of Sydney, Camperdown, NSW 2050, Australia

**Keywords:** KT, Allied health, Evidence-based practice, Online KT tool

## Abstract

**Background:**

It is difficult to foster research utilization among allied health professionals (AHPs). Tailored, multifaceted knowledge translation (KT) strategies are now recommended but are resource intensive to implement. Employers need effective KT solutions but little is known about; the impact and viability of multifaceted KT strategies using an online KT tool, their effectiveness with AHPs and their effect on evidence-based practice (EBP) decision-making behavior. The study aim was to measure the effectiveness of a multifaceted KT intervention including a customized KT tool, to change EBP behavior, knowledge, and attitudes of AHPs.

**Methods:**

This is an evaluator-blinded, cluster randomized controlled trial conducted in an Australian community-based cerebral palsy service. 135 AHPs (physiotherapists, occupational therapists, speech pathologists, psychologists and social workers) from four regions were cluster randomized (n = 4), to either the KT intervention group (n = 73 AHPs) or the control group (n = 62 AHPs), using computer-generated random numbers, concealed in opaque envelopes, by an independent officer. The KT intervention included three-day skills training workshop and multifaceted workplace supports to redress barriers (paid EBP time, mentoring, system changes and access to an online research synthesis tool). Primary outcome (self- and peer-rated EBP behavior) was measured using the Goal Attainment Scale (individual level). Secondary outcomes (knowledge and attitudes) were measured using exams and the Evidence Based Practice Attitude Scale.

**Results:**

The intervention group’s primary outcome scores improved relative to the control group, however when clustering was taken into account, the findings were non-significant: self-rated EBP behavior [effect size 4.97 (95% CI -10.47, 20.41) (p = 0.52)]; peer-rated EBP behavior [effect size 5.86 (95% CI -17.77, 29.50) (p = 0.62)]. Statistically significant improvements in EBP knowledge were detected [effect size 2.97 (95% CI 1.97, 3.97 (p < 0.0001)]. Change in EBP attitudes was not statistically significant.

**Conclusions:**

Improvement in EBP behavior was not statistically significant after adjusting for cluster effect, however similar improvements from peer-ratings suggest behaviorally meaningful gains. The large variability in behavior observed between clusters suggests barrier assessments and subsequent KT interventions may need to target subgroups within an organization.

**Trial registration:**

Registered on the Australian New Zealand Clinical Trials Registry (ACTRN12611000529943).

## Introduction

Cerebral palsy (CP) is the most common physical disability in childhood [1]. Of people with CP, three in four are in pain; one in two have an intellectual disability; one in three cannot walk; one in three have a hip displacement; one in four cannot talk; one in four have epilepsy; one in four have a behavior disorder; one in four have bladder control problems; one in five have a sleep disorder; one in five dribble; one in ten are blind; one in fifteen are tube fed; and one in twenty-five are deaf [2]. Allied health professionals (AHPs) who treat people with CP are therefore faced with complex clinical decision making. Also, like many other fields, new evidence-based CP treatments are rapidly emerging [3]. AHPs provide the majority of health services to these people and therefore need to have up-to-date knowledge and skills in providing evidence-based interventions. AHPs endorse providing evidence-based care [4,5], but goodwill alone does not guarantee the latest research is translated and applied within practice [6,7]. Survey research suggests that there is a significant gap between best available evidence and what treatments are actually offered to people with CP [8,9]. Lack of time [10], lack of skill searching and appraising research [11,12], and lack of access to databases compounded by large volumes of published research are barriers to new knowledge being translated in a timely and efficient way [13].

Knowledge translation (KT) strategies including workshops [14], mentoring [15], outreach visits [16], audit and feedback [17] and reminders and memos [18] aim to embed research into practice and lead to small to moderate changes in health professionals’ behavior. Even though KT is an emergent science, it is known that KT strategies should be tailored to be context specific, and planned in response to a thorough assessment of barriers and facilitators [19,20]. Although there is no firm evidence that multifaceted strategies are more effective than single interventions it is plausible that they would be more effective if each component and the overall strategy were designed in response to a barriers analysis [19]. In the field of CP, a tailored KT intervention was pilot tested with good results, but the lack of a controlled comparison group precludes certainty of the findings [7].

**Figure 1:**
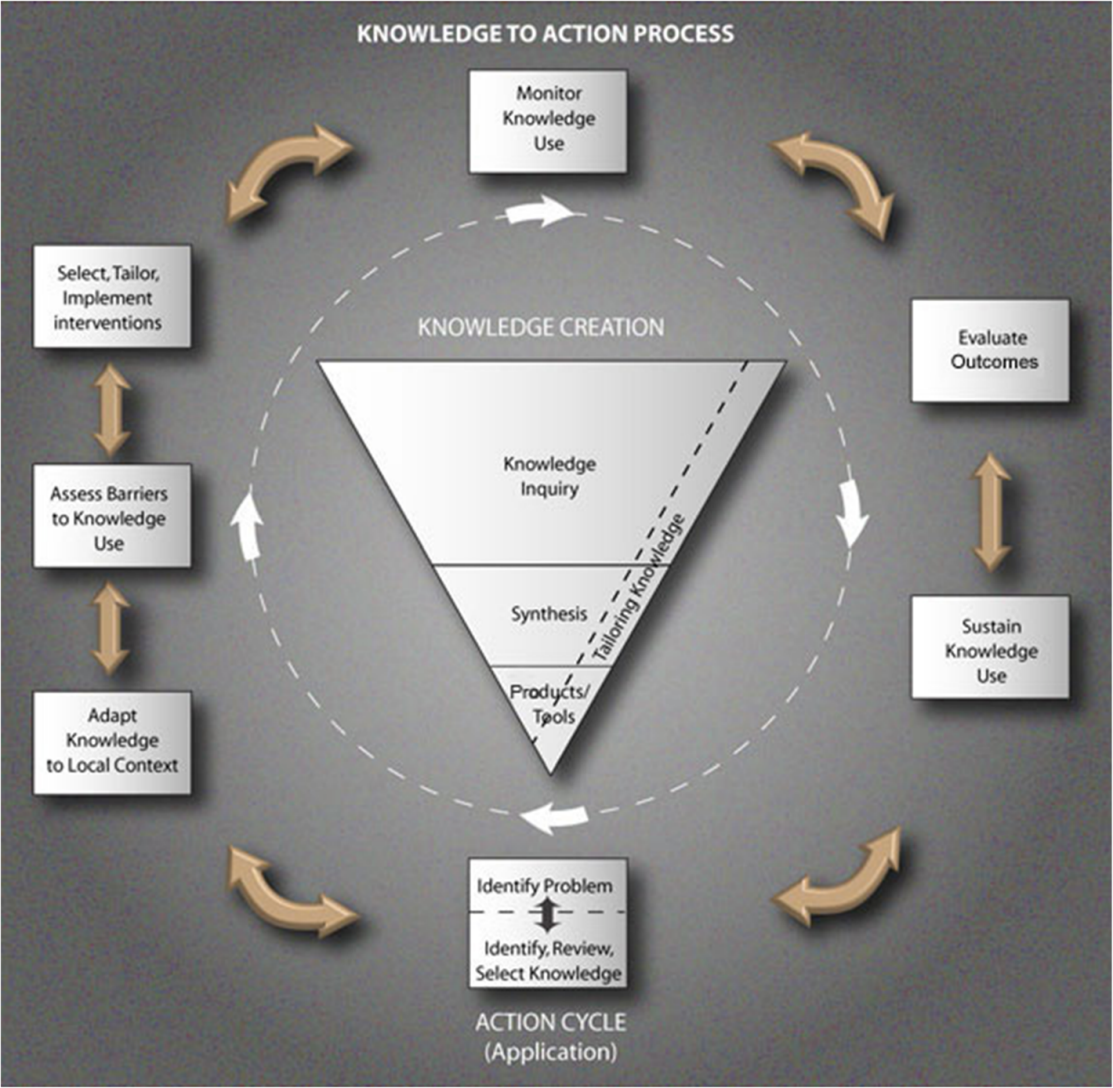
**Knowledge-to-Action (KTA) process.** Source: Graham et al. (2006) [22].

In addition to tailoring KT interventions, it is recommended that theory is used to guide the KT journey [21]. A number of KT frameworks have been proposed, that incorporate key theories suited for various target settings and professional groups. One example is the knowledge-to-action process (KTA) [22] (Figure 1) which provides a comprehensive and flexible framework to guide and monitor a multifaceted KT intervention. Although the use of theory is recommended there are few rigorous studies detailing the application of theory to a KT intervention [23].

**Figure 2:**
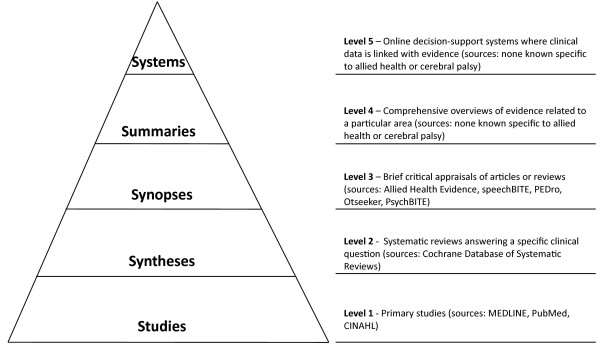
**The 5S pyramid model of evidence-based information resources.** Adapted from Straus & Haynes (2009) [3].

Central to the KTA process, and indeed the basic unit of a KT intervention is up-to-date research being available and accessible to the target group [19,22]. The basis of a KT intervention is synthesis of research in the form of systematic reviews, evidence summaries or online KT tools. Although health professionals generally prefer systematic reviews to original research articles [24], they still report that systematic reviews do no always answer their clinical questions [3,25]. There is an increasing call for customized, easy to read summaries. Straus and Haynes (2009) describe the ‘5S’ model [3,26] for organizing evidence-based information resources (Figure 2). The model is displayed in a pyramid with five levels (studies, syntheses, synopses, summaries, systems) that aim to be increasingly readable, reliable, and relevant as one moves up the pyramid. The top two levels (summaries and systems) may also be referred to as KT tools [19]. Straus and Haynes recommend a top down approach for answering clinical questions.

Previous studies measuring the effectiveness of evidence-based information resources (5S pyramid level 3) detected a change in use however did not detect a change in evidence-based practice (EBP) behavior [27,28]. Dobbins et al. [29] found that targeted messages (5S pyramid level 3 – 4) were more effective than knowledge brokering and access to research evidence for incorporating evidence into public health policies and programs. Although evidence-based information resources are available for AHPs (PEDro, OTseeker, SpeechBite) they are at 5S pyramid level 3 (synopses), and no studies have rigorously evaluated the usefulness of these tools. There are no KT tools (5S pyramid levels 4 or 5) found in literature specifically targeting AHPs working with people with CP.

KT tools presenting up to date research in a user-friendly way, is however only one piece of a KT strategy. Changing EBP behavior is complex as there is a range of behaviors required to be an ‘evidence-based AHP.’ Previous studies have either used self-developed measures [30-33] or have only measured a narrow domain of EBM behavior [34,35]. KT research in the allied health professions measuring EBP behavior across a range of AHPs is also absent from our evidence base [36,37].

The primary aim of this cluster randomized controlled trial (RCT) was to evaluate the effectiveness of a multifaceted KT intervention for improving EBP behavior of AHPs. The central element of the KT intervention was an online evidence-based information resource called the Evidence Alert System (EAS). The EAS contained actionable messages (5S pyramid level 4 and 5), clinical decision-making tools and used the ‘top-down’ approach [3]. The other elements of the multifaceted intervention (workshop, mentoring and documentation changes) reinforced, educated and supported the approach set out in the EAS ensuring that the decision-making tools were embedded into the participant’s workflow. The secondary aims were to measure the effect of the KT intervention on EBP knowledge and attitudes. Our study sought to address key gaps in the current KT evidence by: using an RCT to measure the effect of a multi-component KT intervention centred around the EAS; measuring a wide range of EBP behaviors; and sampling a wide range of AHPs. Aims were measured at the individual participant level. Findings are reported according to the updated CONSORT statement for cluster randomized trials [38] (See Additional file 1).

## Methods

### Trial design and study setting

A multi-site evaluator-blinded, cluster RCT was conducted in a community based CP service in New South Wales (NSW), Australia. NSW is the largest state with a population of approximately 7.25 million people (32% of Australia’s total population). The CP service had 16 sites across NSW, organized into four geographically distinct regions, where AHP services were provided. Each region had centralized management for the sites within its boundaries including clinical seniors, professional development activities and mentoring, and thus was considered a natural cluster grouping. An independent officer not associated with the trial, used computer generated random numbers, to create four opaque envelopes based upon simple randomization. Four geographically distinct clusters were randomized to the intervention or control group. Cluster randomization was chosen to reduce risk of contamination that may have occurred if individuals working at the same site were randomized to different interventions. Individual participants were consented after randomization for pragmatic reasons. The first author (LC) obtained participants’ written consent and data collection took place before and after the workshops, at worksites or nearby locations, between June 2009 and August 2009.

### Ethics

The project was approved by the National Health and Medical Research Council Human Research Ethics Committee at Cerebral Palsy Alliance (Approval number: 2009-05-01) and University of Notre Dame Ethics Committee. The study was registered with Australian New Zealand Clinical Trials Registry (ACTRN12611000529943).

### Participants

**Figure 3:**
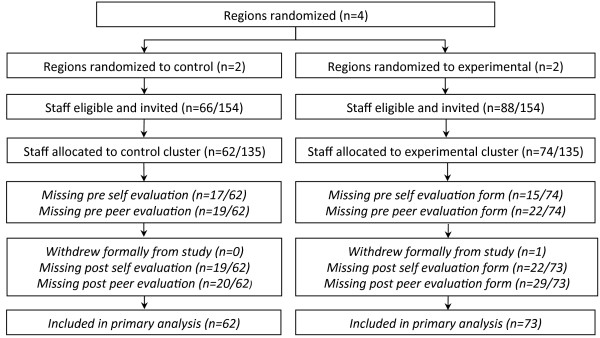
Participant flow diagram for RCT – from randomisation to primary analysis.

Eligible participants were AHPs employed at the study site providing direct clinical services to people with CP and their families. Figure 3 shows the flow of participants through the study. Exclusion criteria were: managers (non-clinical staff); staff without university qualifications; and staff who were not scheduled to work on the day of the workshops.

### Intervention

#### Theoretical model

The theoretical model underpinning the project was the KTA process (Figure 1) developed by KT field leaders [22]. The KTA process first, involves knowledge creation (*i.e.*, production of research syntheses) and second, knowledge application (*i.e.*, identification of the research-practice gap; adaption of the research syntheses to local context; identification of utilization barriers; selection of tailored KT strategies to redress barriers; and monitoring, evaluating and sustaining EBP implementation use). Emerging evidence suggests that KT interventions underpinned by theory may be superior to those that are not theoretical-informed, although more research is needed to confirm this [37]. The advantage of theory-informed KT interventions is that they offer a generalizable framework for other researchers and organizations and provide guidance for designing KT interventions to overcome known barriers [37].

### Assessment of barriers and facilitators

A comprehensive assessment of barriers and facilitators was done over a one-year period. This took the form of meetings between managers, policy makers, researchers, senior clinicians, and knowledge brokers, as well as observation of clinical staff. As there is no firm evidence regarding the superiority of one KT intervention over another [19], researchers and knowledge brokers jointly designed the KT intervention based on whether or not the barrier was modifiable by a pragmatically feasible intervention. Modifiable barriers included lack of skill, time and knowledge. Partially modifiable or non-modifiable barriers were: that evidence was considered not clinically relevant, that staff did not have access to full electronic databases and that some staff had negative attitudes towards EBP. Modifiable barriers, theoretical underpinnings and strategies for the KT intervention are detailed in Table 1. Details of how the components of our multifaceted intervention correspond to the KTA process are shown in Table 2.

**Table 1:** Theoretical basis and strategies to address modifiable barriers

**Barrier: Lack of confidence/skill searching, appraising and synthesizing research evidence**		
**KT intervention**	**Underpinning theory or group of theories**	**Strategy/rationale**
Workshop	Problem based learning, learning styles	Workshops used problem based learning approach and a variety of approaches to ensure that different learning styles were catered to, maximizing the likelihood of increased confidence and skill levels
EAS	Cognitive	Accurate, relevant research evidence on cerebral palsy assessment and treatment was provided via the EAS building skill by modeling synthesis and summary of treatment areas. The EAS bypassed the need for high-level appraisal skills.
Mentoring	Educational	AHPs were included in the problem solving process during mentoring sessions and aimed to increase confidence and build skill base.
	** BARRIER: LACK OF TIME**	
**KT intervention**	**Group of theories that the intervention relates to**	**Strategy/rationale**
EAS	Cognitive	The provision of accurate, relevant research evidence bypassed the need for extensive time spent searching and appraising research via databases and journals.
Paid EBP time in policy	Reimbursement	Paid, protected time for AHPs to engage in EBP activities was provided
	Leadership	Changing policy suggested management ‘buy in’ and endorsement to support changes throughout the organization (leadership theory)
Documentation changes including a reminder system	Total quality management (TQM)	Patient documentation and work processes were reorganized to support clinical decision making and save time (reminder systems, checklists and directing participants to the EAS)
** BARRIER: EVIDENCE CONSIDERED AS NOT CLINICALLY RELEVANT**		
**KT intervention**	**Group of theories that the intervention relates to**	**Strategy/rationale**
Workshop teaching EAS	Educational	AHPs were involved in the problem solving process, so that they ‘owned’ and were a part of the process and could see the applicability of the EAS. Having the 8 week period in between workshops, allowed independent learning and time to apply the EAS information to a real client
	Motivational	Facilitators aimed to convince AHPs of the relevance of research in their area by exploring the EAS through clinical examples and role playing
EAS	Marketing	An appealing product (the EAS) was developed and this was disseminated in a variety of ways (workshop, mentoring, documentation changes)
** BARRIER: NO ACCESS TO FULL ARTICLES AND RESEARCH DATABASES**		
**KT intervention**	**Group of theories that the intervention relates to**	**Strategy/rationale**
EAS	Organizational learning	All staff members at every level of the organization had access to current cerebral palsy evidence and exchange of information via mentoring sessions and team meetings was promoted
** BARRIER: SOME STAFF WITH NEGATIVE ATTITUDES TOWARDS EBP**		
**KT intervention**	**Group of theories that the intervention relates to**	**Strategy/rationale**
Workshop	Social	Credible staff facilitated workshops, modeled positive attitudes and
		emphasized ‘buy in’ from decision-makers in the organization
Mentoring	Social	Mentors were selected with positive attitudes towards EBP so that target behavior was modeled

**Table 2:** KT intervention with corresponding KTA phases

	**WHAT PART OF THE KTA CYCLE DID THE INTERVENTION IMPACT?**					
**KT INTERVENTION**	Creating knowledge	Localising knowledge	Identifying barriers	Redressing barriers	Maintaining use	**WHO IMPLEMENTED IT?**
*Before RCT*						Managers
**Strategic planning meetings**		**•**	**•**	**•**	**•**	Human Resources
						Knowledge brokers
						Policy Makers
**Policy changes (policies developed however not implemented until RCT)**		**•**		**•**		Managers
Provision of paid, dedicated EBP time						Human Resources
Provision of a policy endorsed EBP mentoring program						Knowledge brokers
Mandated and compulsory use of psychometrically sound outcome measures with all clients embedded in workflow *e.g.*, included within mandatory Individual Family Service Plans						Policy Makers
**Evidence alert system development**	**•**					Research Investigators
*During RCT (8-weeks; June – Aug 2009)*						
**Skills training workshops (three-days)**		**•**	**•**	**•**	**•**	Peers
						Knowledge Brokers
**Paid EBP time, mentoring, compulsory use of outcome measures (see policy changes above), documentation changes including reminder systems**		**•**		**•**	**•**	

### Development of multifaceted intervention

**Figure 4:**
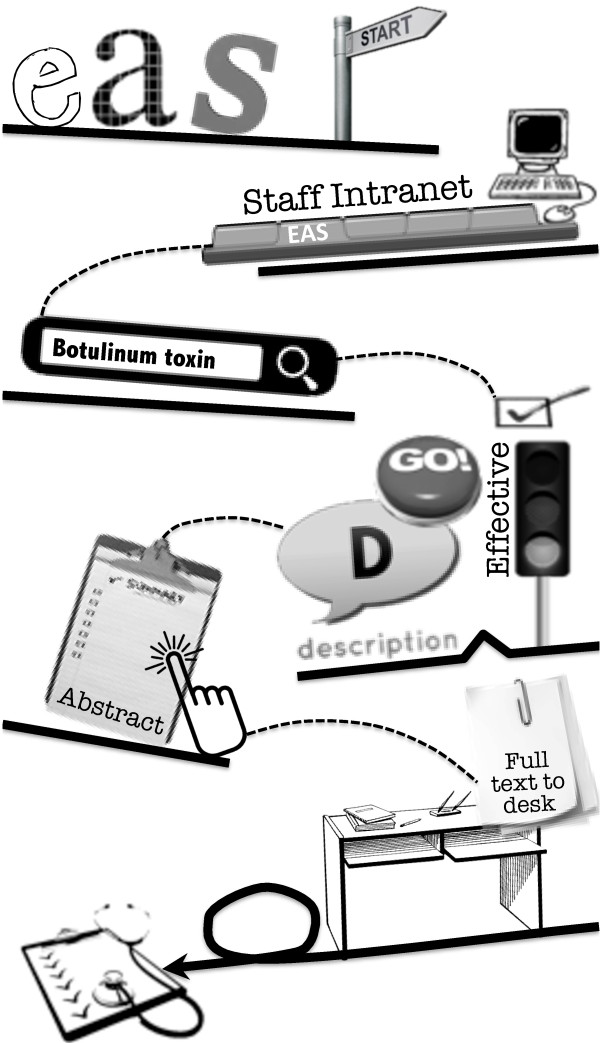
Evidence Alert System infogram.

Strategic planning meetings were held every six weeks in the year leading up to baseline and included researchers, knowledge brokers, policy makers, and managers. Knowledge brokers were senior staff with allied health backgrounds (one per discipline employed in the most senior role for each discipline). Policy makers were the senior executive staff and managers involved in direct management of AHPs in the organization. Goals around EBP behaviors were set and strategies to achieve these goals were jointly selected based on barriers literature and assessment of the study site. The EAS formed the basis of our KT intervention and was developed by research staff and knowledge brokers using freely available software MediaWiki (Figure 4). The EAS included succinct summaries of all the CP research evidence about intervention, prognosis and outcome measurement. Intervention evidence was labeled using the traffic light system [7] where each intervention was given a traffic light color with an actionable message attached. Green = ’go’ if high-quality evidence supports the effectiveness of this intervention, yellow = ’measure’ where low-quality or conflicting evidence supports the effectiveness of this intervention, therefore measure the outcomes of the intervention to ensure the goal is met, and red = ’stop’ where high-quality evidence demonstrates intervention is ineffective, therefore do not use this approach. Decision-making algorithms with embedded evidence summaries were also available on the EAS. Each section of the EAS included abstracts of research articles, descriptions of the intervention or assessment, and a hyperlink to access the full article.

### Experimental group intervention

The intervention group (total n = 73; region A = 39; region B = 34) received a multifaceted KT intervention. A three-day skills training workshop included:

1. Part one, (two days) of the interactive workshop provided training to apply the EAS to decision-making within daily clinical work. A series of clinical examples were explored using the interface of the EAS, training about evidence levels, clinical decision-making algorithms and use of two psychometrically sound, cross disciplinary outcome measures.

2. Part two (one day) of the workshop eight weeks later involved participants presenting a case study detailing how they used the EAS to inform their clinical decision making with a real patient. This was followed by discussion with a small group of colleagues designed to help participants demonstrate the integration of their learning into their own clinical work.

Investigators and each senior clinician [39] led the workshops using knowledge brokering strategies [40]. There was a mix of instructional techniques including didactic, interactive, role-playing and reflection. There was collaboration within and between professional groups.

On day-1 of the 3-day workshop, participants were informed that they had access to the EAS and that there were policy changes including: paid, quarantined EBP time; changes to client documentation including reminders to use the EAS; and embedding outcome measurement within workflow and mentoring by knowledge brokers.

The KT intervention was directed at the cluster level (three-day workshop-part one, access to the EAS and policy changes) and individual level (mentoring, and three-day workshop, part two). Details of the KT intervention are shown in Table 2.

### Control group

The control group (total n = 62; region C = 29, region D = 33) received an equal intensity intervention about communication skills with no EBP content and no use of the EAS: three-day workshop about AHP-client communication skills and workplace supports (paid communication time, strategic planning, mentoring) related to communication skills. To minimize the risk of contamination, the control group was not informed about the EAS, paid EBP time, knowledge brokers, or mentoring until the end of the trial. The changes to documentation were not implemented in the control group clusters until the end of the RCT.

### Outcome measures

#### Primary outcome

The primary endpoint was change in self- and peer-rated EBP behavior from baseline to eight weeks (individual and cluster level) measured using Goal Attainment Scaling (GAS) [41]. Participants rated themselves against the self-GAS scales, and then to limit measurement bias, in a separate environment, a well-acquainted peer rated their performance on the peer-GAS scales. Selection of the GAS instrument increased study rigor because it overcame known instrumentation limitations in the KT literature surrounding EBP behavior measurement, including: responsivity—GAS has established validity, reliability, and exquisite responsivity to change, whereas systematic review evidence indicates that for nearly all valid and reliable EBP instruments, test responsivity is unknown [42]; tailoring—GAS is an individualized measure of change, and so progress towards any target behavior (including health professional behaviors [43]) could be validly, reliably, and sensitively measured, including tailored EBP behaviors unique to the study site, *e.g.*, notifications to the CP Register; comprehensive measurement – GAS is an individualized measure of change, and so we could comprehensively measure all desired EBP behaviors, whereas systematic review evidence indicates that other psychometrically sound EBP instruments measure knowledge instead of behavior, or are limited because they only measure one discrete aspect of EBP behavior [29,42,44-46]; and lack of gold standard tool—accurate, gold-standard, flawless measurement of EBP behavior is not yet established in literature [47]. Even though direct observation of EBP behavior (such as simulated patients, video/audio recordings of practice) is perceived as methodologically preferable to indirect (proxy) reports of EBP behavior (such as chart audit, patient report, self-report, or peer-report), systematic review evidence indicates that direct measures often fail validity testing [47]. This could have introduced other flaws to our clinical trial. Moreover, collecting direct measures throughout NSW, being a state-wide service, would have introduced prohibitive trial costs (NSW’s landmass is 3.25 times larger than the United Kingdom, and is larger than California and New Mexico combined), when the cost-benefit of a potentially invalid measure is weighed-up. Even though self-report proxy measures are an imperfect measure of actual behavior [47], leading KT agencies, such as the Canadian Institutes of Health Research advocate for self-report because the process of self reflection plays a critical role in initiating behavioral changes within organizations. In light of current EBP behavior measurement limitations, GAS offered the best way forward since it was psychometrically sound, it comprehensively measured EBP behavior, was practical across an entire state and could be tailored to the study site.

The GAS scales were devised by a multidisciplinary panel of experts familiar with EBP behaviors of the eligible AHPs, as per literature recommendations for scale establishment. Twenty-five goal scales were developed, one-half relating to EBP behaviors and the other one-half relating to communication behavior for the control group. The scales measured EBP behaviors such as: use of gold standard goal-setting tools to plan services; use of CP classification systems to accurately prognosticate; use of evidence (*e.g.*, via the EAS) to quickly choose evidence-based classification systems, interventions and outcome measures; and use of gold standard outcome measures to routinely evaluate services. The GAS scales are available from the corresponding author by request. As per the test manual, raw scores were converted to GAS T-scores, enabling inferential statistical analysis of continuous data.

### Secondary outcomes

Self- and peer-rated attitude changes were measured using subsets three and four of the Evidence-Based Practice Attitude Scale (EBPAS) [48], which is psychometrically permissible. EBP knowledge was measured via open-ended exam questions with right/wrong answers, pre-defined by the panel of experts, derived from published evidence.

EAS utilization was measured by number of web page hits collected via a software program that tracked cluster-specific IP addresses in batches. Web hit data collection was concealed from participants, minimizing the likelihood of observer bias affecting EAS use.

### Adverse events

An adverse event log was not required because the intervention was educational in nature and therefore posed no risk.

### Blinding

Blinding was judiciously applied wherever pragmatically possible, resulting in a single-blinded trial. This included: independent evaluator blinding to group allocation and phase of the trial when scoring outcome data; and partial participant and facilitator blinding to the specific EBP behavior of interest to the investigators. Participants and workshop facilitators were clearly aware of the content of the workshops, however were not aware of which intervention (KT intervention or communication skills) was of specific interest to the researchers. Fidelity of the evaluator blinding was not formally investigated.

### Sample size

We sought to test the efficacy of an organizational KT intervention and therefore conducted the study within one agency, which is the largest of its kind in Australia. This methodological decision imposed pragmatic limitations on the obtainable sample frame. We successfully recruited 88% of the available sampling frame, however the total number of employees at the agency was less than the number of participants required to reach statistical power if correlation of outcome variables within sites was observed (intra-cluster correlation). A sample size calculation identified the probability of detecting an effect size of 1 at an alpha level of 0.05 (one-tail) and a power of 90%. For Goal Attainment Scaling [mean T-score = 50, standard deviation (sd) = 10] an improvement of 10 points or more in the KT intervention group than the control group was sought, (improvement of 1 sd). The expert panel agreed that a 10-point increase in GAS T-scores equated to significant clinical improvement in EBP behaviors. The calculation assumed a 20% non-consent rate and a 20% attrition rate indicating a sample size requirement of 72 (38 per group) for a non-cluster trial. We enrolled 135 professionals (n = 73 interventions and n = 62 controls) at four sites. Based on estimating an intra-cluster correlation co-efficient (ICC) of 0.1 we calculated that the study was underpowered to demonstrate an improvement of 10 points between groups if a cluster effect of this size was observed (Variance Inflation Figure = 4.3).

### Statistical analysis

All statistical analysis was carried out with individual participants as the unit of analysis on an intention-to-treat basis by using SPSS for Windows 19.0.0 (SPSS Inc, Chicago, IL) and SAS 9.3 (SAS Institute, Cary NC).

We conducted generalized linear regression analysis for primary and secondary endpoints, using post intervention GAS T-score as the outcome variable and adjusting for potential confounding variables (baseline GAS T-score, profession, group allocation, grade level, and years in the disability field). Effect sizes with 95% confidence intervals (CIs) were calculated and significance was set at 0.05. These estimates would underestimate the standard errors and confidence intervals for the effect size if participant outcomes are correlated within cluster sites, thus mixed effects models with cluster included as a random effect were used to adjust for a cluster effect to calculate the effect size for each outcome [49]. ICC was calculated from the mixed effects model and bootstrapping (1,000 samples generated) was performed to calculate 95% confidence intervals for the ICC.

## Results

A total of 135 AHPs (n = 73 interventions and n = 62 controls) were recruited (see Figure 3), which was 88% of the available sampling frame. At baseline, participant attributes were mostly comparable between groups, the exception being prior EBP education attendance (88% compared to 66% for controls) (Table 3). To account for this baseline difference, prior EBP education was treated as a covariate in the regression model. Included professionals were physiotherapists (24%), speech pathologists (26%), occupational therapists (37%), psychologists (6%), and social workers (7%). 64% of participants had over five years experience working with people with disabilities although 63% of the cohort had worked at the study site for less than five years. 94% of the sample had English as their first language. The return rate for the GAS and EBPAS ratings were between 60% and 82% (see Figure 3), with the primary end-point having more missing data. The KT intervention group had 19/73 (31%) eight-week GAS forms missing, compared to the control group who had 17/62 (30%). This difference between groups was not statistically significant (chi square p = 0.95).

**Table 3:** Baseline characteristics of participants

	**KT Intervention**	**Control**
	**n = 73 (%)**	**n = 62 (%)**
**Professional background**		
Occupational therapist	23 (31)	26 (42)
Physiotherapist	16 (22)	16 (26)
Speech pathologist	20 (27)	16 (25)
Psychologist	7 (10)	1 (2)
Social worker	7 (10)	3 (5)
**Grade level**		
Level 1	19 (26)	14 (23)
Level 2 (clinical specialist)	34 (47)	37 (60)
Level 3 (clinical senior)	13 (18)	8 (13)
Manager or other	7 (9)	2 (3)
**Years’ experience in disability field**		
<2 years	11 (15)	16 (26)
2-4 years 11 months	10 (14)	12 (19)
5-9 years 11 months	25 (34)	14 (23)
>10 years	27 (37)	20 (32)
**Previous EBP continuing education?**		
Yes	64 (88)*	41 (66)*
No	9 (12)*	21 (34)*

*Significant difference between groups at baseline therefore treated as a covariate in the analysis.

### Clustering effect

The ICC for the primary endpoints were 0.33 (95% CI 0.16,0.69) for self-rated GAS T-scores, that is 33% of the total variation observed in self-rated GAS T-scores can be attributed to differences between the sites, (rather than differences between individuals within each site), and 0.64 (95% CI 0.36,0.80) for peer-report GAS T-scores (Table 4), that is 64% of the total variation observed peer-rated GAS T-scores can be attributed to differences between sites. These results demonstrate the correlation of GAS T-scores within sites was very large, whereas there was a large variation in scores between sites. This cluster effect substantially depleted the study power (because participant scores within each site cannot be regarded as independent). ICCs were smaller for secondary outcomes (Table 4).

**Table 4:** Primary and secondary outcomes

			**Treatment n = 73**		**Control n = 62**		**Base model**			**Mixed effects model**	
	**Outcome**		n*	**Mean (sd)**	n*	**Mean (sd)**	**Difference (95% CI)**	**p**	**ICC (95% CI)**	**Difference (95% CI)**	**p**
EBM Behavior	Self	Baseline	59	54.05 (13.80)	45	55.42 (10.92)					
		8-weeks	51	65.96 (13.49)	43	62.45 (19.50)	5.08 (0.40,10.55)	0.07	0.33 (0.16,0.69)	4.43 (-10.63,19.49)	0.56
	Peer	Baseline	52	61.83 (13.69)	43	61.52 (16.95)					
		8-weeks	44	74.26 (8.51)	42	68.41 (16.63)	7.86 (1.97,13.75)	0.01	0.64 (0.36,0.80)	6.75 (-16.95,30.44)	0.57
EAS page hits**				6,123		1,677					
EBM Knowledge		Baseline	57	7.91 (3.05)	50	8.09 (3.52)					
		8-weeks	52	10.69 (2.23)	45	8.02 (3.13)	3.29 (2.25,4.33)	<0.0001	0.01 (0.0,0.26)	3.29 (2.18,4.40)	<0.0001
EBP attitude EBPAS	Self subset 3	Baseline	55	2.67 (0.75)	47	2.57 (0.70)					
		8-weeks	50	2.63 (0.74)	44	2.77 (0.61)	-0.27 (-0.57,0.03)	0.08	0.0 (0.0,0.32)	-0.27 (-0.57,0.03)	0.08
	Subset 4	Baseline	55	3.00 (0.51)	47	2.98 (0.58)					
		8-weeks	50	3.03 (0.61)	44	2.98 (0.59)	0.03 (-0.22,0.28)	0.82	0.0 (0.0,0.25)	0.03 (-0.22,0.28)	0.82
	Peer subset 3	Baseline	42	2.93 (0.63)	38	2.90 (0.72)					
		8-weeks	32	3.17 (0.56)	39	1.17 (0.80)	0.03 (-0.37,0.42)	0.88	0.0 (0.0,0.51)	0.03 (-0.37,0.43)	0.88
	Subset 4	Baseline	42	0.89 (0.78)	32	3.19 (0.61)					
		8-weeks	32	0.87 (0.75)	32	1.13 (0.93)	-0.23 (-0.75,0.23)	0.37	0.12 (0.0,0.65)	-0.29 (-1.06,0.48)	0.45

*Number of participants who completed outcome measure.

**EAS page hit raw data could only be collected and analyzed at the cluster level, not the individual level because the electronic data was collected in batches.

### Effectiveness of KT intervention

#### Primary outcome—EBP behaviors

Self-rated GAS T-scores increased more in the intervention group compared to controls however this difference was not statistically significant after adjusting for the cluster effect; Effect size 4.43 [95% CI -10.63 to 19.49 (p = 0.56)] (Table 4). Baseline self-rated GAS T-scores were a predictor in the model [Effect size 0.71 (95% CI 0.52–0.90) (p < 0.0001)]; indicating lower performers improved but remained lower performers, and higher performers improved and remained leading performers. No other covariates were significantly predictive of outcome.

Peer-rated GAS T-scores of the intervention group also increased compared to controls, but this difference was also not statistically significant after adjusting for the cluster effect: effect size 6.75 [95% CI -16.95 to 30.44 (p = 0.57)] (Table 4). Similar to the self-rated GAS T-scores, the final peer-rated GAS T-score was predicted by the baseline peer-rated GAS T-score [effect size 0.30 (95% CI 0.150.45) (p < 0.0001)]. No other covariates were significantly predictive of peer-rated GAS T-scores. The peer-rated GAS T-scores for each cluster mirrored the self-rated GAS cluster T-scores, suggesting the observed study effects were behaviorally meaningful, despite low study power to demonstrate a statistically significant difference.

### Secondary outcomes—EBP knowledge and attitudes

EBP knowledge scores increased compared to controls, with a statistically significant effect size of 2.97 (95% CI 1.97, 3.97, p < 0.0001). The ICC for this outcome was zero, and this effect remained statistically significant after adjusting for the cluster effect: 2.97 (95% CI 1.97, 3.97, p < 0.0001). Baseline score (p < 0.0001) and professional category (p = 0.03) were also predictors in the model. There was minimal to no correlation between participants within sites for self- or peer-rated EBP attitudes, however we did not demonstrate a statistically significant intervention effect (Table 4). The intervention group accessed the EAS more than the control group (KT intervention group 6,123 total hits; control group 1,677 hits).

Secondary analyses examining mean outcome scores for each cluster revealed that both clusters in the KT intervention group improved their self- and peer-rated GAS T-scores as expected (Table 5). One of the control group clusters (cluster 3) also responded as expected, with very minimal increases in self- and peer-rated GAS T-scores from baseline to eight weeks (self-rated T-score change = 0.22; peer-rated T-score change = 2.27). The other control group cluster (cluster 4) had high baseline scores (self –rated GAS T-score = 66.41; peer-rated GAS T-score = 73.32) and further improved by 10.15 points over the 8-week study period, despite not receiving the KT intervention (Table 5). We performed *post hoc* Spearman’s correlation tests to assess for correlation between knowledge and attitude scores (at baseline, 8-weeks and change scores) overall, by treatment group, and within individual clusters. No statistically significant positive correlations were found.

**Table 5:** Mean outcome scores for each cluster

**Variable**			**Outcome score N, mean (sd) per cluster**			
**Outcome**		**Time**	**Cluster 1 (Exp)**	**Cluster 2 (Exp)**	**Cluster 3 (Control)**	**Cluster 4 (control)**
**EBP behavior**	**Self GAS**	**Baseline**	35	24	28	17
			50.73 (13.75)	58.88 (12.64)	48.75 (10.85)	66.41 (15.46)
		**8-weeks**	24	27	22	21
			66.39 (16.02)	65.58 (11.08)	48.97 (15.34)	76.56 (11.92)
	**Peer GAS**	**Baseline**	33	19	28	15
			60.19 (14.26)	64.68 (12.51)	55.20 (15.69)	73.32 (12.57)
		**8-weeks**	21	23	23	19
			72.69 (9.93)	75.69 (6.90)	57.47 (13.11)	81.66 (9.05)
**EBP knowledge**	**Exam score**	**Baseline**	35	22	28	22
			7.69 (2.76)	8.27 (3.51)	6.50 (3.08)	10.11 (3.04)
		**8-weeks**	25	27	23	22
			10.80 (2.37)	10.59 (2.14)	6.98 (3.26)	9.11 (2.65)
**EBP attitude**	**Self EBPAS subset 3 score**	**Baseline**	35	20	27	20
			2.73 (0.73)	2.57 (0.79)	2.53 (0.61)	2.64 (0.83)
		**8-weeks**	24	26	22	22
			2.55(0.78)	2.70 (0.70)	2.52 (0.57)	3.01 (0.55)
	**Self EBPAS subset 4 score**	**Baseline**	20	35	27	20
			2.86 (0.48)	3.08 (0.54)	2.84 (0.56)	3.16 (0.58)
		**8-weeks**	24	26	22	22
			3.10 (0.59)	2.96 (0.64)	2.85 (0.60)	3.11 (0.58)
	**Peer EBPAS subset 3 score**	**Baseline**	30	12	23	15
			2.80 (0.60)	3.24 (0.63)	2.87 (0.74)	2.95 (0.73)
		**8-weeks**	16	16	17	15
			3.20 (0.47)	3.14 (0.65)	3.07 (0.63)	3.32 (0.57)
	**Peer EBPAS subset 4 score**	**Baseline**	30	12	23	16
			0.83 (0.64)	1.03 (1.08)	1.45 (0.86)	0.77 (0.48)
		**8-weeks**	16	16	17	15
			1.05 (0.86)	0.69 (0.60)	1.41 (0.99)	0.82 (0.76)
**Web hits**	**Page hits**	**8-weeks**	2987	3136	928	749

## Discussion

We conducted a cluster RCT to evaluate whether a multifaceted KT strategy changed AHP’s EBP behaviors. Both clusters in the KT intervention group improved within the study period, but not statistically significantly more than the control group. We consider this null finding to be a probable type II error because our study was underpowered owing to the fact that the number of participants required to account for clustering of EBP behaviors within sites exceeded the number of employees available. Our study demonstrated increased use of our evidence-based resource (the EAS), however we were unable to confirm that this translated to a statistically significant change in EBP behavior. This finding is in line with previous research involving evidence-based resources [27,28]. Owing to the type II error, we remain unsure of the true effect of our KT intervention, but we discovered a number of potentially important findings that may contribute to future KT endeavours and the body of research.

The high ICCs (ranging from 0.33 to 0.64) for EBP behavior measures, indicated substantial correlation of behaviors within clusters, and indicated differences in behaviors between clusters. When we examined the mean change scores for each cluster, one of the four clusters (cluster 4), which was randomly allocated to the control group, was an obvious outlier with high baseline GAS T-scores, high baseline knowledge scores and increased self- and peer-rated GAS T-scores over the study period.

Variability between natural groupings (such as clinical, departmental or regional) has been noted in the KT literature previously [29,32]. Perhaps the high baseline EBP scores for the cluster 4 reflected positive EBP culture and practices due to cluster 4’s manager [32,50,51]. The notion that a manager can strongly influence research culture is by no means new [29,52], as some opinion leaders are known to strongly influence EBP behavior [50,53]. The cluster 4’s manager was active in promoting EBP behavior among staff. A large range of KT interventions were in place in cluster 4 prior to this study, including audit and feedback, financial incentives, workshops and mentoring. It is conceivable that cluster 4 therefore had both better readiness and receptivity to EBP supports as they had essentially been engaging in active KT for a longer period than the other clusters [32]. That said, positive EBP culture is considered to be related to positive EBP attitudes [52] and EBPAS scores measuring attitude change of cluster 4 were no different from the other clusters at baseline or eight weeks. This may have reflected measurement error, or may indicate that positive attitudes in cluster 4 were not necessary as mandatory policies within that cluster were the driving force behind the higher GAS scores.

### Secondary outcomes

Our hypothesis that the KT intervention would improve knowledge was supported with the KT intervention group knowledge exam scores showing a statistically significant improvement compared to the control group. This finding supports previous research suggesting that knowledge change alone does not consistently translate into behavior change [7,32,37,54]. Interestingly, change in knowledge scores was not affected by the cluster effect suggesting that knowledge is not as susceptible to peer influences as behavior.

We found no correlation between behavior, knowledge, and attitude change scores within and between clusters. Attitudes remained unchanged. We hypothesise the lack of change in EBP attitudes in our study may be explained by: (1) high baseline EBP attitudes and there was conceivably a ceiling effect on the EBPAS. This was plausible as EBP had been a focus in the organization for some time prior to the RCT. In this case, positive attitudes at baseline, increased knowledge scores and policy changes may together have resulted in the behaviorally meaningful changes observed. There is however no normative data for AHPs on the EBPAS, so it is difficult to say whether or not baseline attitudes were high compared to AHPs in other organisations; (2) EBPAS subsets potentially not being sensitive enough to detect attitude change and the psychometrics for sensitivity in this population are unknown; (3) the EBPAS being an accurate, sensitive measure and that attitudes did not improve from the KT intervention. This third possibility supports the notion that improved knowledge was not adequate to lead to statistically significant behavior change, and that a shift in attitudes was also needed [55]. Conversely, the behaviorally significant change that was observed potentially bypassed the need for attitude change by employing strategies such as mandatory use of documentation and outcome measures; and (4) EBP attitudes taking a longer period of time than knowledge to change, and the eight-week trial was too short to detect change.

### Strengths and limitations

The study had a number of strengths including the rigorous design and broad robust behavior measurement. Our chosen measurement instrument (GAS) was sensitive to change [56,57] and appeared accurate as self- and peer-rated scores mirrored each other. Distinguishing features of our study were that we measured a wide set of behaviors among AHPs working with people with CP. The mix of AHPs in our sample is fairly representative of other community based disability organizations, increasing external validity. This is the first RCT in the KT literature involving social workers, psychologists or occupational therapists [37]. The KT intervention itself was a study strength being based on a solid theoretical model [21-23], in response to a comprehensive barriers assessment, with desired outcomes clearly defined, and included a range of interventions, not only educational interventions [37].

There are a number of study limitations. First and foremost, the pragmatic constraints that limited the number of available clusters and participants led to low statistical power causing a probable type II error. Second, the large differences observed between clusters suggest that we potentially should have tailored the KT intervention to each cluster rather than the whole organization. Third, the evidence base regarding whether proxy behavior measures represent actual behavior is not firmly established, but with preferred rival direct measures also lacking validity and reliability [41,58]. Moreover, direct measurement was not affordable in our study given the geography involved, and indirect measurement tools were therefore used [43,59]. To minimize measurement bias, systematic review recommendations regarding indirect measures were followed, and included using: acceptable indirect measures [41,59] (such as self- and peer-rated behavior triangulated with unbiased web hit data) [42]; measurement tools with strong psychometric properties [47]; more than one tool to measure behavior change [47]; and a sound theoretical model as a basis of the intervention [21]. The time frame of the trial was short considering that many EBP behaviors and system/organizational changes (such as documenting client goals and mentoring) take time to develop [60]. A follow-up study is needed to measure whether the EBP behaviors were sustained [14]. The return rate of the GAS exam form and EBPAS was not perfect (60-82%), with the eight-week data having more missing data.

## Conclusions

KT literature recommends tailoring KT interventions to overcome known barriers within organizations [19,20], however our findings suggest that this may need to go even further with KT interventions being designed for subgroups within an organization. The impact of different workplace culture may mean that there are dramatically different barriers needing different KT interventions to be effective [32]. Considering the importance of management-led change, targeting policy makers and managers may be beneficial. This has been done in the public health sector [29], however no studies customizing KT to policy makers/management was found in the allied health literature. Our study provides extremely rich pilot study data to planning and conducting an adequately powered cluster RCT in future.

Our study highlighted the methodological challenges of conducting empirical research in a community-based organization with fixed cluster and participant numbers. Whether or not RCTs are a feasible option in community organizations is debatable, and it may be that other research designs are more appropriate [29,61]. Researchers, policy makers, and clients need to effectively collaborate to ensure that reliable, relevant research becomes embedded into everyday care in a timely way. Considering that the cornerstone of KT is access to reliable research, the authors plan to make the EAS publically available.

## Competing interests

The authors declare that they have no competing interests.

## Authors’ contribution

The study was carried out as part of a Doctor of Philosophy candidature by LC IN, SM and staff at the Research Institute of Cerebral Palsy Alliance (the study site) assisted in study design and developing the Evidence Alert System (searching databases for articles, synthesising results, converting the information to electronic format). LC, IN, LM and staff from the Research Institute and senior staff at Cerebral Palsy Alliance facilitated the workshops that formed part of the KT interventions (experimental and control groups). The participants of the study were all staff at the Cerebral Palsy Alliance. All authors had full access to all of the data, including statistical reports and tables and take responsibility for the integrity of the data and accuracy of the data analysis. All authors read and approve the final manuscript.

## Supplementary Material

Additional file 1: Table S1 CONSORT 2010 checklist of information to include when reporting a cluster randomised trial. **Table S2**: Extension of CONSORT for abstracts 1, 2 to reports of cluster randomised trials. Click here for file
